# Evidence of Human Milk Oligosaccharides in Cord Blood and Maternal-to-Fetal Transport across the Placenta

**DOI:** 10.3390/nu11112640

**Published:** 2019-11-04

**Authors:** Birgit Hirschmugl, Waltraud Brandl, Bence Csapo, Mireille van Poppel, Harald Köfeler, Gernot Desoye, Christian Wadsack, Evelyn Jantscher-Krenn

**Affiliations:** 1 Department of Obstetrics and Gynecology, Medical University of Graz, 8036 Graz, Austria; birgit.hirschmugl@medunigraz.at (B.H.); waltraud.brandl@medunigraz.at (W.B.); bence.csapo@medunigraz.at (B.C.); gernot.desoye@medunigraz.at (G.D.); christian.wadsack@medunigraz.at (C.W.); 2 BioTechMed-Graz, 8010 Graz, Austria; mireille.van-poppel@uni-graz.at (M.v.P.); harald.koefeler@medunigraz.at (H.K.); 3 Institute of Sport Science, University of Graz, 8010 Graz, Austria; 4 Core Facility Mass Spectrometry, Center for Medical Research, Medical University of Graz, 8036 Graz, Austria

**Keywords:** Human Milk Oligosaccharides (HMOs), placenta, placental transport, secretor status, pregnancy, fetal circulation, 2′-fucosyllactose

## Abstract

Human milk oligosaccharides (HMOs) are present in maternal serum in early gestation, raising the question of whether HMOs can cross the placental barrier and reach fetal circulation. Here, we aimed to detect HMOs in cord blood, and assess HMO composition and concentration in relation to maternal HMOs. In an ex-vivo placental perfusion model, we asked whether HMOs can pass over the placenta. Using HPLC, we measured HMOs in maternal serum and matching venous cord blood samples collected at delivery from normal pregnancies (*n* = 22). To investigate maternal-to-fetal transport, we perfused isolated placental cotyledons from term pregnancies (*n* = 3) with 2’-fucosyllactose (2′FL) in a double closed setting. We found up to 18 oligosaccharides typically present in maternal serum in all cord serum samples investigated. Median total cord blood HMO concentration did not differ from the concentration in maternal serum. HMO composition resembled the composition in maternal serum, with the strongest correlations for 2′FL and LDFT. After 180 min perfusion, we found 22% of maternally offered 2′FL in the fetal circuit without reaching equilibrium. Our results provide direct evidence of HMOs in cord blood, and suggest that the placenta transfers HMOs from the maternal to fetal circuit. Future studies will investigate potential differences in the transfer of specific HMOs, or in pregnancy disorders.

## 1. Introduction

Human milk oligosaccharides (HMOs) are a complex blend of bioactive glycans in breast milk, offering a variety of potential benefits to the breast-fed neonate [1]. These include prebiotic effects on beneficial bacteria and anti-adhesive/anti-microbial functions against pathogens. Additionally, a growing body of evidence suggests that HMOs also have systemic effects, including protection from allergies, autoimmune diseases, metabolic disorders, or respiratory and urinary tract infections [2,3,4,5,6,7,8,9,10]. HMOs have been shown to reach circulation and can be detected in the blood [11,12], and urine [13,14,15,16] of breast-fed infants. In vitro and in vivo studies showed HMOs can modulate immune functions [3,4], inflammation [5,7,17,18], and metabolic functions [19], supporting the emerging paradigm of a crucial role for HMOs as signal molecules in the systemic circulation.

The more than 150 different HMO structures described [20] all comprise lactose, which can be elongated by galactose/N-acetyl-glucosamine disaccharide units and/or modified by sialic acid and/or fucose residues. As the structure of an HMO determines its function, the relative abundance of individual structures may shape the resulting effect of a complex HMO mixture [21].

In human milk, HMO concentration and composition vary inter-individually, and with lactation stage (intra-individually) [22,23,24,25,26]. Part of the inter-individual variation is explained by genetic polymorphisms in two genes, the *Secretor* and the *Lewis* gene, encoding for two distinct fucosyltransferases, fucosyltransferase-2 (FUT2) and fucosyltransferase-3 (FUT3) [27,28,29]. Individuals with an inactive FUT2 have a negative secretor status and lack α1-2 fucosylated structures, such as 2′-fucosyllactose (2′FL). In turn, the presence of 2′FL is indicative of a positive secretor status. In addition to genetic factors, an emerging body of evidence suggests that environmental factors, such as geographic location and maternal nutritional status, also influence HMO concentration and composition [30,31].

Recently, we showed that HMOs are present in maternal serum already during pregnancy [32]. HMO concentration and composition varied with secretor status and gestational age. 2′FL seemed to be induced between the end of the first trimester and mid-pregnancy, contributing to an increase in total HMOs in secretor-positive women. In another study on overweight and obese pregnant women, we confirmed the presence of HMOs in early pregnancy, and the increase in HMO concentration over the course of gestation. In these previous studies, we found individual HMOs associated with adiposity (measured as subcutaneous fat mass), or with maternal glucose/insulin metabolism [33], suggesting that maternal metabolic status in pregnancy influences serum HMOs. The finding of HMOs in the maternal circulation in pregnancy begged the question of whether HMOs can also reach fetal circulation, potentially affecting the placenta and fetus. Thus, here we hypothesize that HMOs are present in cord blood, and can be transported across the placenta.

To test this hypothesis, we analyzed HMO concentration and composition in cord blood in comparison to maternal serum HMOs at delivery, in a small pregnancy/birth cohort. Moreover, we investigated maternal-to-fetal 2′FL transfer across the human placenta, using an ex-vivo placental perfusion approach.

## 2. Materials and Methods

### 2.1. Study Overview

Subjects were selected from a prospective, observational, longitudinal study of 51 healthy pregnant women, recruited at the Department of Obstetrics, Medical University of Graz, between February and October 2013 (ClinalTrials.gov ID NCT03277807). The original study included the collection of serum samples at three time points during pregnancy (visit 1, at 10–14 gestational weeks, visit 2, at 20–24 gestational weeks, and visit 3, at 30–35 gestational weeks) and at time of admission to the hospital for delivery. Immediately after delivery of the placenta, blood was collected from the umbilical vein. The exclusion criteria of the original study were gestational age greater than the 14th week of gestation at visit 1, multiple pregnancy, more than three consecutive miscarriages, fetal anomalies, diabetes type 1 or 2, pre-pregnancy hypertension, preeclampsia/HELLP (hemolysis, elevated liver enzymes, low platelets), and self-reported smoking. The selection criteria for this study were the absence of gestational pathologies developed during pregnancy, and the availability of maternal serum samples at the time of delivery and corresponding cord blood samples, resulting in a final sample size of 22 women–infant dyads. The study complied with the Declaration of Helsinki guidelines as revised in the year 2000 and was approved by the ethical committee of the Medical University of Graz (#26-380 ex 13/14). All subjects provided written informed consent.

### 2.2. Serum Samples

Venous blood samples from healthy pregnant women were collected after admission to the hospital and before giving birth. Venous cord blood samples were collected by punctuation from the umbilical cord vein. After centrifugation at 3500× *g*, serum samples were stored at −80 °C until analysis.

### 2.3. Human Milk Oligosaccharide Standards

2′-Fucosyllactose (2′FL), 3-fucosyllactose, lacto-*N*-tetraose (LNT), Lacto-*N*- neotetraose (LNnT), lacto-*N*-fucopentaose 1, 2, and 3 (LNFP 1, 2, and 3), Lacto-*N*-difucohexaose 1 (LNDFH1), and Lacto-*N*-hexaose (LNH) were purchased from Dextra Laboratories, Reading, United Kingdom. Lactodifucotetraose (LDFT), 3′-sialyllactose (3′SL), 6′-sialyllactose, 3′SLN, 6′SLN, and (LSTa, b and LSTc and DSLNT (Glycoset II) were purchased from Prozyme, Hayward CA. C13-labelled 2′FL was purchased from Elicityl, Crolles, France.

### 2.4. HMO Isolation and Analysis by HPLC

Oligosaccharides were isolated from serum samples, as previously reported [32]. In brief, serum samples with added internal standard were worked up by removing lipids, proteins, and salts using Chloroform/MeOH and solid phase extraction (SPE). Recovered, dried HMOs from serum samples were fluorescently labelled with 2-aminobenzamide (2AB) [8]. The 2AB-glycans were separated by HPLC with fluorescence detection on a TSKgel Amide-80 column (Tosoh Bioscience, Tokyo, Japan) [32]. Standard retention times were used to annotate HPLC peaks. The amount of each individual HMO was calculated based on pre-determined response factors. The relative abundance of each of the individual HMOs was determined by setting the sum of the 18 identified oligosaccharides as 100% total HMOs. HMO peak annotation by comparison with the retention times of commercially available HMO standards was confirmed by exoglycosidases and LC-MS [32].

### 2.5. Analysis of Human Milk Oligosaccharides by Enzymatic Digest

To confirm the identity of individual peaks in the serum, 2AB-labelled samples were either treated with α2-3-neuraminidase (New England Biolabs, #P0743L) or α1-2-fucosidase (New England Biolabs, #P0724S) according to the manufacturer’s instructions. Enzyme-treated and untreated samples were run in parallel and analyzed by HPLC with fluorescence detection, as described above.

### 2.6. Determination of Oligosaccharides by LC-MS/MS

Maternal serum or venous cord blood samples were prepared as described for the HPLC method and pooled (*n* = 10). An Accela HPLC (Thermo Fisher Scientific) was used with a TSKgel Amide-80 (Tosoh Bioscience, Japan) and a linear gradient of a 50 mmol/L-ammonium formate/acetonitrile solvent system. Oligosaccharides were determined by a TSQ Quantum Ultra (Thermo Fisher Scientific, Waltham, MA, USA) triple quadrupole instrument in positive ESI mode. The spray voltage was set to 4000 V, capillary voltage to 35 V, and vaporizer temperature was 250 °C. Scan width was 1 Da with 0.1 s scan time for each transition.

### 2.7. Ex-Vivo Perfusion of a Single Human Placental Cotyledon

Placentas from C-sections of uncomplicated term pregnancies (*n* = 3) were used after receiving written informed consent. The perfusion method used in this study was previously described by Perazollo et al. [34]. Briefly, closed circuit perfusion was applied with a flow rate of 9 mL/min at the maternal circulation and 3 mL/min at the fetal circulation (250 mL perfusion media). C13-labelled 2′FL (5 μg/mL) was offered at the maternal side. Samples were taken from the maternal and fetal circulation at 5 min, 15 min, and 30 min, followed by 30 min steps for three hours. Perfusion samples (50 μL) were worked up by removing lipids, proteins, and salts using Chloroform/MeOH and SPE. Isolated HMOs were fluorescence-labeled and subsequently analyzed by HPLC with fluorescence detection.

### 2.8. Statistical Analysis

Concentrations of individual and total oligosaccharides were normalized to the internal standard raffinose and expressed as medians and range in pmol/mL [32]. Relative abundances were calculated as the percentage of an individual HMO of the summed unambiguously identified oligosaccharides (total HMOs). The HMO 3FL was excluded from all calculations due to a co-elution with an unidentified peak.

Differences between maternal and fetal HMO concentrations were assessed with the Wilcoxon test for skewed distributed variables. Correlation studies were performed using Spearman correlation (for skewed data) or Pearson analysis for relative abundances. Concentrations of individual and total oligosaccharides were calculated as median and interquartile range (IQR). Statistical analysis was performed using SPSS (version 23) (IBM SPSS, Chicago, IL, USA). Statistical significance was assumed when *p* < 0.05.

## 3. Results

### 3.1. HMO Profiles in Cord Blood Serum Resemble Peripartal HMO Profiles in Maternal Serum

Using an HPLC-based method, we have previously shown that circulating HMOs are detectable in the serum of pregnant women. Here, we aimed to assess whether HMOs are also present in cord blood (fetal HMOs), and to compare maternal HMOs with fetal HMOs at the time of delivery. From the original longitudinal pregnancy study, we had 22 mother–infant dyads with matching specimens (maternal serum and cord blood samples) from the time of delivery. Table 1 shows the main maternal and infant characteristics of the study group (*n* = 22).

**Figure 1:**
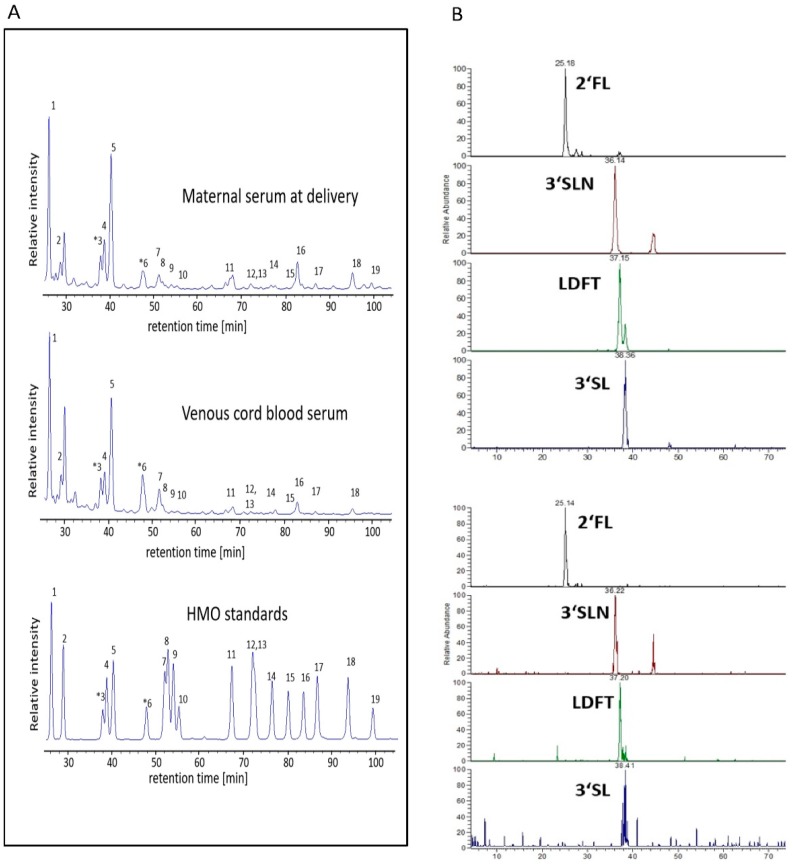
Human milk oligosaccharides (HMOs) are present in cord blood and resemble HMO profiles in maternal serum at delivery. (**A**) Representative HPLC chromatograms of oligosaccharides in pregnant serum at delivery (first panel) and in venous cord blood serum (second panel). As control, a mixture of commercially available human milk oligosaccharide standards (third panel) is shown. (**B**) Mass chromatograms of oligosaccharides isolated from pooled maternal peripartal samples (upper panel) and from pooled cord blood samples (lower panel), acquired by LC-MS/MS in multiple reaction monitoring (MRM) mode. Panels show the mass transitions 609/301 for 2′FL and 3FL, 754/301 for 3′SL, 755/301 for LDFT, 795/325 for 3SLN. (1) 2′-Fucosyllactose (2′FL), (2) 3-fucosyllactose (3FL), (3) 3′-siallyllactosamine (3′SLN), (4) lactodifucotetraose (LDFT), (5) 3′-sialyllactose (3′SL), (6) 6′-sialyllactosamine (6′SLN), (7) 6′-sialyllactose (6′SL), (8) lacto-*N*-tetraose (LNT), (9) lacto-*N*-neotetraose (LNnT), (10) 3′-sialyl-3-fucosyllactose (3′S-3FL), (11) lacto-*N*-fucopentaose 1 (LNFP1), (12,13) lacto-*N*-fucopentaose 2/3 (LNFP2/3), (14) sialyllacto-*N*-tetraose a (LSTa), (15) sialyllacto-*N*-tetraose b (LSTb), (16) sialyllacto-*N*-tetraose c (LSTc), (17) lacto-*N*-difucohexaose (LNDFH), (18) lacto-*N*-hexaose (LNH), (19) disialyllacto-*N*-tetraose (DSLNT).

The HPLC analysis of cord serum revealed the presence of up to 16 HMOs (2′FL, 3FL, 3′SL, 6′SL, LNT, LNnT, 3′S-3FL, LNFP1, 2, 3, LSTa, b, c, LNDFH, LNH, DSLNT) which were also found in maternal serum peripartum. Representative HPLC chromatograms of HMOs isolated from maternal serum and from venous cord serum (Figure 1A) show a strong resemblance of respective HMO profiles, with 2′FL and 3′SL as the two most abundant HMOs. HPLC plots of all samples are provided in the supplementary material. In cord blood serum, we also found the lactosamines 3′SLN and 6′SLN, two oligosaccharides present in maternal serum during pregnancy but not usually present in human milk.

To confirm the identity of the most prominent HMOs in cord blood serum, we performed exoglycosidase digest on samples previously analyzed by HPLC. Treated with α1-2 fucosidase, α2-3 neuraminidase, or β-galactosidase, HPLC profiles revealed removal or major reduction of the expected peaks (2′FL, LDFT, LNFP1 and LNDFH, 3′SL and 3′SLN, and LNT) (data not shown). Using LC-MS, we confirmed the respective masses for most abundant peaks (2′FL, 3′SLN, LDFT, and 3′SL) assigned by the retention times of standards (Figure 1B).

### 3.2. HMO Concentration and Composition in Mother–Infant Dyads

**Figure 2:**
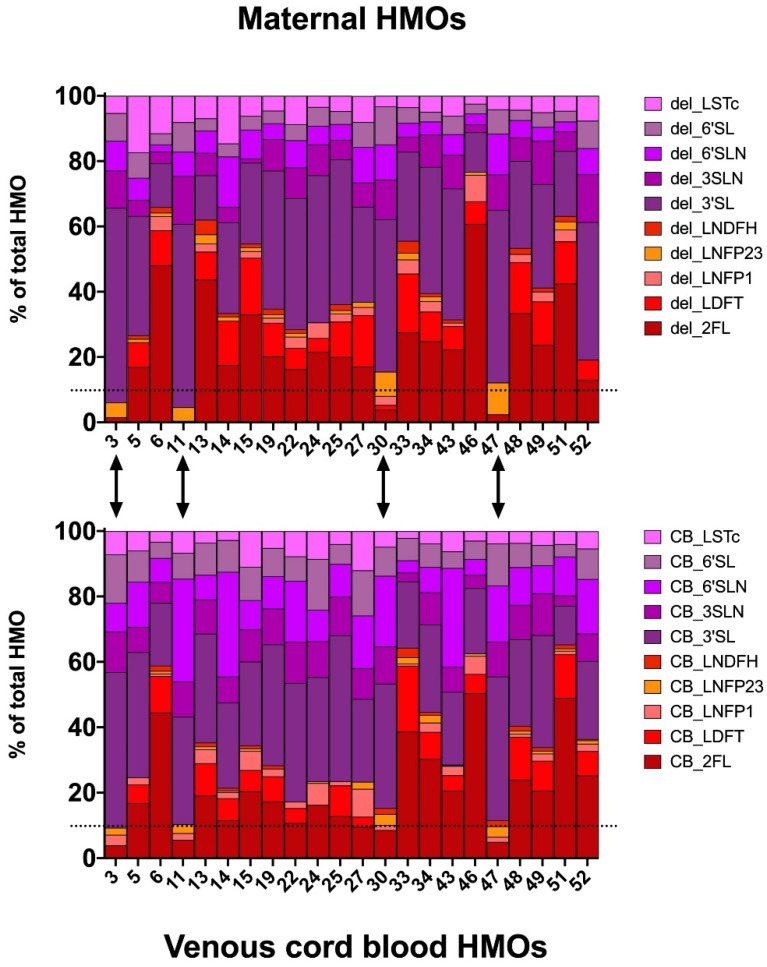
Relative HMO concentrations in maternal serum (**upper panel**) and cord blood serum (**lower panel**) allows for assignment of the secretor status. Stacked bar plots showing the percentage of total HMOs in 22 mother–infant pairs. 2′FL percentage below 10% indicates secretor-negative status and assigned status matches between maternal and cord blood samples. Dotted line marks the 10% of 2′FL cut-off used to discriminate between secretor positive and negative individuals. Arrows depict the four secretor-negative sample pairs (#3, #11, #30, and #47).

The median concentrations (with IQR) of total HMOs at delivery were found to be 611.0 pmol/mL (480.5–911.3) in maternal serum peripartum, and 870.9 pmol/mL (360–1225.3) in serum from umbilical veins (Table 2). We found significant differences between maternal and fetal samples in the concentrations of 6′SLN and 6′SL (Wilcoxon test, *p* < 0.05), but not in other individual HMOs, grouped, or total HMOs. HMO composition in cord blood resembled the composition of HMOs in respective matching maternal serum samples (Figure 2). For 4 out of the 22 mothers (18%), we assigned a negative secretor status based on a relative 2′FL abundance below 10%, and this assigned secretor status was consistent with the secretor status assigned based on fetal HMOs in venous cord blood.

### 3.3. HMOs in Venous Cord Blood Associate with Maternal HMOs

**Figure 3:**
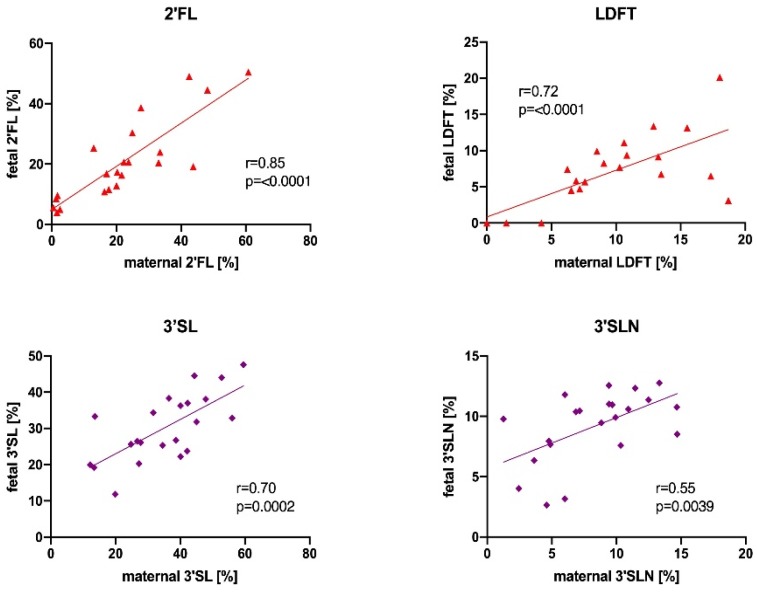
Relative HMO concentration in maternal serum peripartum correlate with fetal HMOs in cord blood. Scatter plots with Pearson correlation are shown for 2′-fucosyllactose (2′FL), lactodifucotetraose (LDFT), 3′-sialyllactose (3′SL), 3′-siallyllactosamine (3′SLN) (*n* = 22).

To get an insight into the origin of fetal HMOs, we investigated associations between HMO concentrations in cord blood and maternal serum. We found that relative abundances of individual HMOs were highly correlated between maternal and venous cord blood. Figure 3 shows correlations between maternal and fetal samples for relative concentrations of 2′FL, LDFT, 3′SL, and 3′SLN (additional correlation plots are shown in Supplementary Figure S1). Absolute concentrations of the secretor-active HMOs, 2′FL and LDFT, were correlated between maternal and fetal samples (Spearman *r* = 0.49, *p* = 0.0095, and *r* = 0.62, *p* = 0.011, respectively), while sialylated HMOs, 3′SL and 3′SLN, did not significantly correlate (data not shown). We also tested whether absolute or relative HMO concentrations in venous cord blood differed based on infant sex, but found no difference in venous cord blood HMOs.

### 3.4. Maternal to Fetal 2′FL Transport across the Placenta

**Figure 4:**
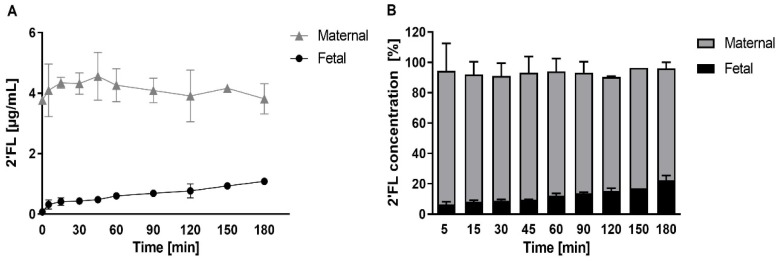
Maternal to fetal 2′FL transfer across the placenta. Ex-vivo placenta perfusion experiments (*n* = 3) were performed in presence of 2′FL (5 µg/mL) in the maternal circulation. (**A**) 2′FL concentrations (± SD) were determined in maternal and fetal circulation after 5, 15, 30, 45, 60, 90, 120, 150, 180 min by HPLC. (**B**) 2′FL recovery was calculated in maternal and fetal samples at distinct time points. Data are expressed as percentage (± SD) of initial concentration in the maternal circulation.

Finding a strong correlation for the α1,2-fucosylated 2′FL between maternal and fetal circulations in vivo, and thus, matching secretor status for mother–infant dyads, we next asked whether the placenta can transport 2′FL from the maternal to the fetal side. To investigate this, we used an ex-vivo perfusion model of the human placenta. After a washout phase (1 h), we perfused isolated cotyledons with 2′FL (5 µg/mL) in the maternal reservoir in a double closed setting for 180 min. We observed a time-dependent 2′FL transfer from maternal to fetal circulation and detected 2′FL in the fetal vein already within 5 min of perfusion. Mean concentration increased from 0.37 µg/mL to 1.1 µg/mL (7% and 22% of the offered 2′FL concentration, respectively), without reaching an equilibrium after 180 min. We did not detect 2′FL in samples taken after the initial washout phase as well as from the fetal reservoir at the start of perfusion (Figure 4A). Approximately 20% of offered 2′FL from the maternal side was recovered in the fetal circuit (Figure 4B).

## 4. Discussion

Here, we provide strong evidence that HMOs are also present in the serum of the umbilical vein, and thus, in the fetal circulation. Additionally, we found associations between fetal HMOs in cord blood and HMOs in maternal serum at delivery, especially for HMOs associated with a positive secretor status. Using an ex-vivo human placental perfusion model, we demonstrated that 2′FL is transported from the maternal to the fetal circuit. This result corroborates the maternal to fetal correlation of absolute 2′FL concentrations found in the paired cohort. Together, these findings indicate a first contact of the infant with HMOs already in utero, and point towards a common origin of maternal and fetal HMOs in the maternal compartment.

### 4.1. HMO Concentration and Composition in the Maternal and Fetal Circuits

Using an HPLC-based approach, we report the same HMO species in cord serum as identified in maternal serum in late pregnancy, and confirm the identity by exoglycosidase digest and mass spectrometry. The concentrations of total or specific HMOs in cord serum were not significantly different compared to maternal serum at delivery, except for 6′SL and 6′SLN, which were higher in the fetal circulation. At present, we cannot explain the observed differences in these rather low abundant oligosaccharides. Possible reasons may range from a different metabolism via sialyltransferases and accumulation in the fetal circulation, to an alternative origin of these particular oligosaccharides. However, since the majority of the specific HMOs did not significantly differ between maternal and fetal circulations, a common origin seems plausible. Accordingly, HMO composition in the fetal compartment resembled the composition in maternal serum at delivery. The lactosamines 3′SLN and 6′SLN, which are usually not found in human milk, were integral components of the fetal HMO profiles, consistent with our current and previous findings on HMOs in maternal serum in pregnancy [32,33,35].

We have previously described temporal variations in maternal serum HMO concentration and composition throughout pregnancy [32,33]. Since the fetal circulation is usually not accessible during an ongoing pregnancy, here we observe a snapshot of ‘fetal HMOs’ at the time point of delivery, and can only speculate about the quality and quantity of exposure to HMOs during fetal life. However, investigating amniotic fluid from amniocentesis at 17–20 weeks of gestation, we have some preliminary evidence that the same HMO species identified in cord blood are also present in the fetal compartment earlier in pregnancy (Jantscher-Krenn, unpublished). Accordingly, a recent study reported on four HMOs (2′FL, 3FL, LDFT, and 6′SL) in amniotic fluid collected at delivery [36]. HMOs are known to be excreted by breast-fed infants [15]. Along this line, it is tempting to speculate that HMOs in amniotic fluid may originate from the fetal circulation. The fetus might excrete circulating HMOs via urine, once the fetal kidneys have started working, leading to the accumulation of HMOs in amniotic fluid.

### 4.2. Biological Roles of Fetal HMOs

Our findings not only confirm HMOs as integral components of the intra-uterine environment, but also point towards a novel direct role of HMOs in the (fetal) circulation, merely because of their presence. In breast-fed infants, less than 1% of the HMOs offered via human milk are thought to be taken up from the gut into the systemic circulation [15], and some HMOs were detected in infant blood [11,12] and urine [13,14,15,16]. Given the high HMO concentration in human milk, the primary roles of HMOs seem to lie in their local prebiotic [37,38], anti-infective [39,40,41], or immunomodulatory effects, mainly in the gut [2,42]. There, the effects can be indirect (microbiome dependent), or direct, targeting host cells (microbiome independent). However, in recent years, HMOs have increasingly received attention as signal molecules with effects on various cells and tissues [3,4,7,17,18,19,43]. Our finding of HMOs in the cord blood and, thus, in the systemic circulation of the developing fetus, seems to strongly enforce this notion of biological roles for HMOs as signal molecules. The systemic circulation, and thus, endothelial cells, immune cells, or fetal liver cells, might be the first targets of HMOs in utero. Fascinatingly, the fetus seems to be in contact with HMOs inside and out, not only bathing in HMO-containing amniotic fluid, but also being thoroughly ‘permeated’ by circulating maternally derived HMOs, potentially months before birth.

### 4.3. HMO Transport across the Placenta

The similar composition of maternal and fetal HMOs at delivery, and the strong correlation of specific HMOs between the maternal and fetal compartment, indicate that HMOs have a common origin and are transported across the placenta. Our results from the ex-vivo 2′FL perfused placenta strongly support this notion.

Fucosylated HMOs in absolute and relative concentrations strongly correlated between maternal and infant circuits, while absolute sialylated HMOs did not. This might speak for different transport mechanisms between fucosylated and sialylated HMOs. It will be interesting to study if, and how, the placenta regulates the transport of specific HMOs across the barrier. Assuming a common origin of prenatal HMOs in the mother, i.e., in the mammary gland, HMOs in the fetal circulation might not only depend on maternal serum concentration and composition of HMOs with different charge and polarity, but potentially also on (changing) placental transport capacity during gestation. The full-term placenta transferred approximately 20% of offered 2′FL from maternal to fetal circuit without reaching equilibrium after 180 min perfusion of the tissue. The transfer of 2′FL followed a linear kinetic, slow clearance, and the total recovered 2′FL of both circuits almost gained 100%. Strikingly, these findings suggest a direct trans-placental passage of this glycan without being metabolized or trapped in the tissue. The underlying mechanisms of 2′FL uptake, its transport across the tissue, and its efflux to the fetal circulation remain elusive. Future studies will compare the transfer of specific HMOs (fucosylated, sialylated, or unmodified), and investigate whether transport mechanisms can be altered in pregnancy disorders, such as gestational diabetes and preeclampsia, with implicating effects on placental and fetal development.

### 4.4. Modifiers of Fetal HMOs: Maternal and Fetal Factors

Recent studies suggest that maternal metabolic status influences HMO concentration and composition [31,44]. We recently showed that prenatal maternal 2′FL negatively correlated with subcutaneous body fat [32], and in another study, found 3′SL associated with maternal glucose parameters [33]. However, the relatively small sample size in this pilot study did not allow for investigating the associations between maternal metabolic factors and fetal HMOs.

### 4.5. Gestational Age

If maternal HMOs determine fetal HMOs, gestational age could be a factor affecting fetal HMOs. We have previously found maternal HMOs in serum to be strongly dependent on gestational age, with the largest differences found in α1,2-fucosylated HMOs between early and mid-pregnancy [32]. Thus, it would be interesting to investigate whether (early) preterm infants have a different HMO cord blood profile than term infants. In a study on preterm labor, we analyzed maternal HMOs at the time of admission with suspected preterm labor between 24–34 weeks of gestation, and found higher maternal serum 3′SL concentration to be associated with lower gestational age at birth [35]. However, here, in this relatively small cohort, all infants were born at term. Whether the mode of delivery, and the associated labor in the case of a natural birth versus a secondary C-section, has an impact on the HMO profile in the mother and/or the fetus also remains to be investigated. In our cohort, the vast majority of the women either had a spontaneous birth, or underwent a secondary C-section after they had labor, but could not naturally deliver their babies. Only two women had primary C-sections due to medical reasons.

### 4.6. Infant Factors

We did not find any sex specific differences in HMO concentration or composition in cord blood. While this may also be due to the limited sample size, it is in line with previous studies on HMOs in human milk [31]. This, again, speaks more for an origin of fetal HMOs in the maternal compartment.

### 4.7. Secretor Status

Secretor-associated α1-2-fucosylated HMOs were highly correlated, whereas sialylated HMOs showed weaker or no correlations between mother and infant. Assigned secretor status based on the presence of 2′FL did not reveal any mismatches. The finding that fetal 2′FL correlates with maternal 2′FL concentration, and that maternally offered 2′FL is transported across the placenta ex vivo, are good indications that HMOs in cord blood stem from a maternal origin.

## 5. Conclusions and Significance

Our study provides direct evidence of HMOs in cord blood, and suggests that the placenta transfers HMOs from the maternal to fetal circuit, identifying HMOs as key factors in a healthy intrauterine environment. These observations may fundamentally change our view of the role of human “milk” oligosaccharides, reinforcing HMOs as pregnancy-associated factors, present also in the fetal compartment and potentially critical for placental and fetal development. The novel concept that HMO composition may affect not only the health of the breast-fed newborn, but already have implications on the health of mother and feto-placental unit will set the stage for future research. The placenta, both as an organ of transport, and as target tissue, needs to be studied further within this context.

## Figures and Tables

**Table 1:** Maternal and fetal characteristics of study population.

Maternal and Infant Characteristics	Total *n* = 22	
	Mean	± SD
Maternal Age (years)	34.6	4.5
BMI (kg/m^2^) pre-pregnancy	21.9	2.8
BMI delivery (kg/m^2^)	27.9	3.2
Weight gain (kg)	16.3	5.8
Parity primiparous (*n*, %)	14 (63.6)	
Mode of delivery, vaginal (*n*, %)	15 (68.2)	
Gestational age at delivery (d)	278	7.8
Infant sex (male *n*, %)	11 (50)	
Ponderal index	2.45	0.22
Infant Birth weight (g)	3324	297
Placental weight (g)	496.3	149.0

**Table 2:** HMO concentration in maternal serum at delivery and in cord blood (pmol/mL).

	Maternal Serum (*n* = 22)	Fetal Serum (*n* = 22)	*p* Value (Wilcoxon Test)
HMO	Median (IQR)	Median (IQR)	
2′FL	124.7 (43.3–236.6)	111.41 (40.0–286.4)	0.485
3′SLN	41.4 (30.9–59.6)	69.4 (26.5–97.1)	0.178
LDFT	66.4 (30.4–95.7)	39.8 (7.1–91.1)	0.260
3′SL	215.1 (163.8–279.2)	244.9 (95.6–309.8)	0.961
6′SLN	39.2 (30.7–47.1)	101.7 (27.4–196.1)	0.001
6′SL	33.2 (23.8–45.9)	70.0 (21.9–97.4)	0.006
LNT	n.q.	n.q.	
LNnT	n.q.	n.q.	
LNFP1	12.0 (0.0–24.6)	15.6 (10.1–26.9)	0.615
LNFP2/3	9.6 (3.8–17.0)	7.3 (1.5–23.2)	0.858
LSTc	38.4 (24.8–57.2)	42.8 (19.1–56.2)	0.758
LNDFH	5.9 (0.0–11.9)	2.33 (0.0–12.6)	0.723
DSLNT	9.5 (1.8–16.4)	n.q.	
fucosylated	212.6 (94.5–375.3)	181.4 (86.6–435.0)	0.615
sialylated	409.3 (283.1–532.3)	657.7 (175.5–756.6)	0.178
Total HMO	611.0 (480.5–911.3)	870.9 (360–1225.3)	0.322

## Supplementary Materials

The following are available online at https://www.mdpi.com/2072-6643/11/11/2640/s1.

## Abbreviations

HMO	Human Milk Oligosaccharide
2′FL	2′-fucosyllactose
3FL	3-fucosyllactose
LNT	lacto-*N*-tetraose
LNnT	lacto-*N*-neo-tetraose
LNFP	lacto-*N*-fucopentaose
LNDFH	lacto-*N*-difucohexaose
LNH	lacto-*N*-hexaose
LDFT	lactodifucotetraose
3′SL	3′-sialyllactose
6′SL	6′-sialyllactose
3′SLN	3′-siallyllactosamine
6′SLN	6′-siallyllactosamine
LST	sialyllacto-*N*-tetraose
DSLNT	disialyllacto-*N*-tetraose
SAT	subcutaneous adipose tissue
FUT2	fucosyltransferase-2
FUT3	fucosyltransferase-3
