# Supplementary material for: Evidence of Human Milk Oligosaccharides in Cord Blood and Maternal-to-Fetal Transport across the Placenta
Source: Nutrients. 2019 Nov 4;11(11):2640. doi: 10.3390/nu11112640 (PMC6893815; doi:10.3390/nu11112640)
Supplement: Supplementary file 1 [file nutrients-11-02640-s001.pdf]

nutrients

## Supplementary Material

# Evidence of Human Milk Oligosaccharides in cord blood and maternal to fetal transport across the placenta

Birgit Hirschmugl[1,2], Waltraud Brandl[1], Bence Csapo[1], Mireille van Poppel[2,3], Harald Köfeler[2,4], Gernot Desoye[1], Christian Wadsack[1,2], Evelyn Jantscher-Krenn[1,2]*

[1]Department of Obstetrics and Gynecology, Medical University of Graz, Graz, Austria; [2]BioTechMed-Graz, Austria; [3]Institute of Sport Science, University of Graz, Graz, Austria; [3]Core Facility Mass Spectrometry, Center for Medical Research, Medical University of Graz, Graz, Austria

**Running title:** HMOs in cord blood

**Keywords:** Human Milk Oligosaccharides (HMO); placenta; placental transport; secretor status; pregnancy; fetal circulation; 2'-fucosyllactose

**Word count:** 3952

***Correspondence:** Evelyn Jantscher-Krenn, PhD; ORCID ID 0000-0003-3568-891X

Department of Obstetrics and Gynecology, Medical University of Graz, Auenbruggerplatz 14/2, 8036 Graz, Austria

Tel +43 316 385 80076

Fax +43 316 385 12506

Email evelyn.jantscher-krenn@medunigraz.at

This contains

1) HPLC plots of HMOs isolated from all investigated maternal serum and cord serum samples

2) Supplementary Figure 1 showing correlation plots of relative concentrations of HMOs

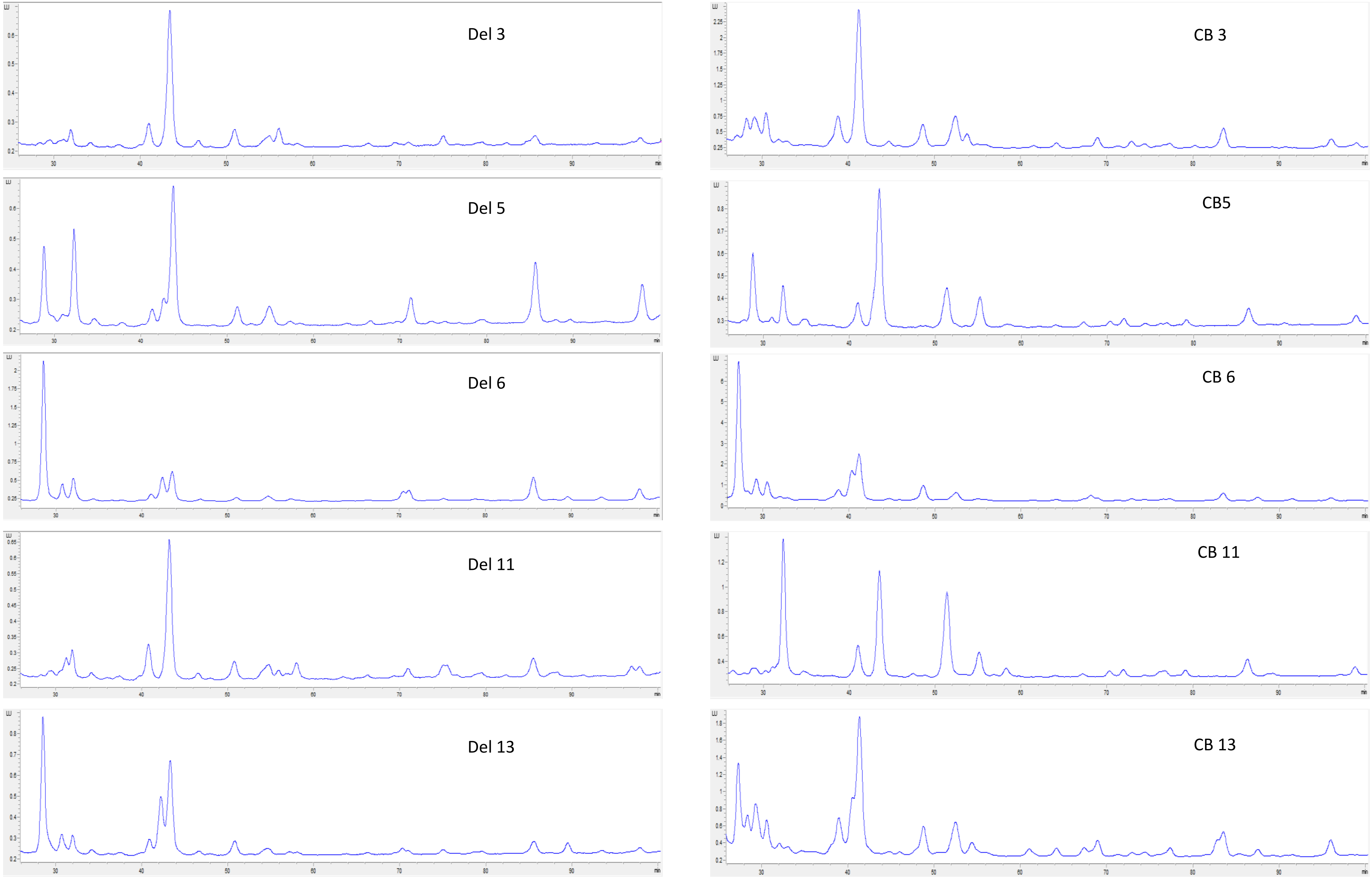
Del 3
CB 3
Del 5
CB5
Del 6
CB 6
Del 11
CB 11
Del 13
CB 13

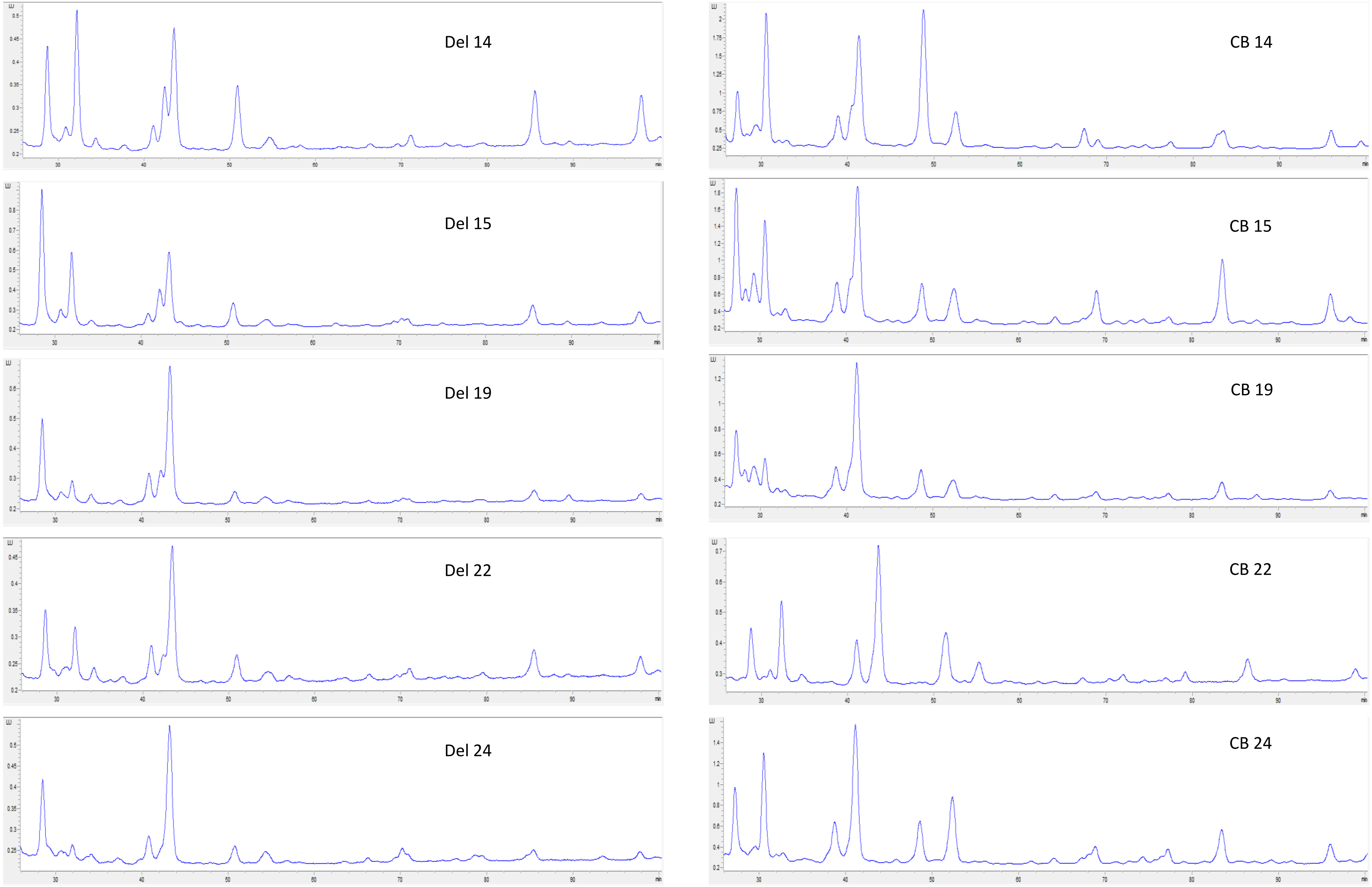
Del 14
CB 14
Del 15
CB 15
Del 19
CB 19
Del 22
CB 22
Del 24
CB 24

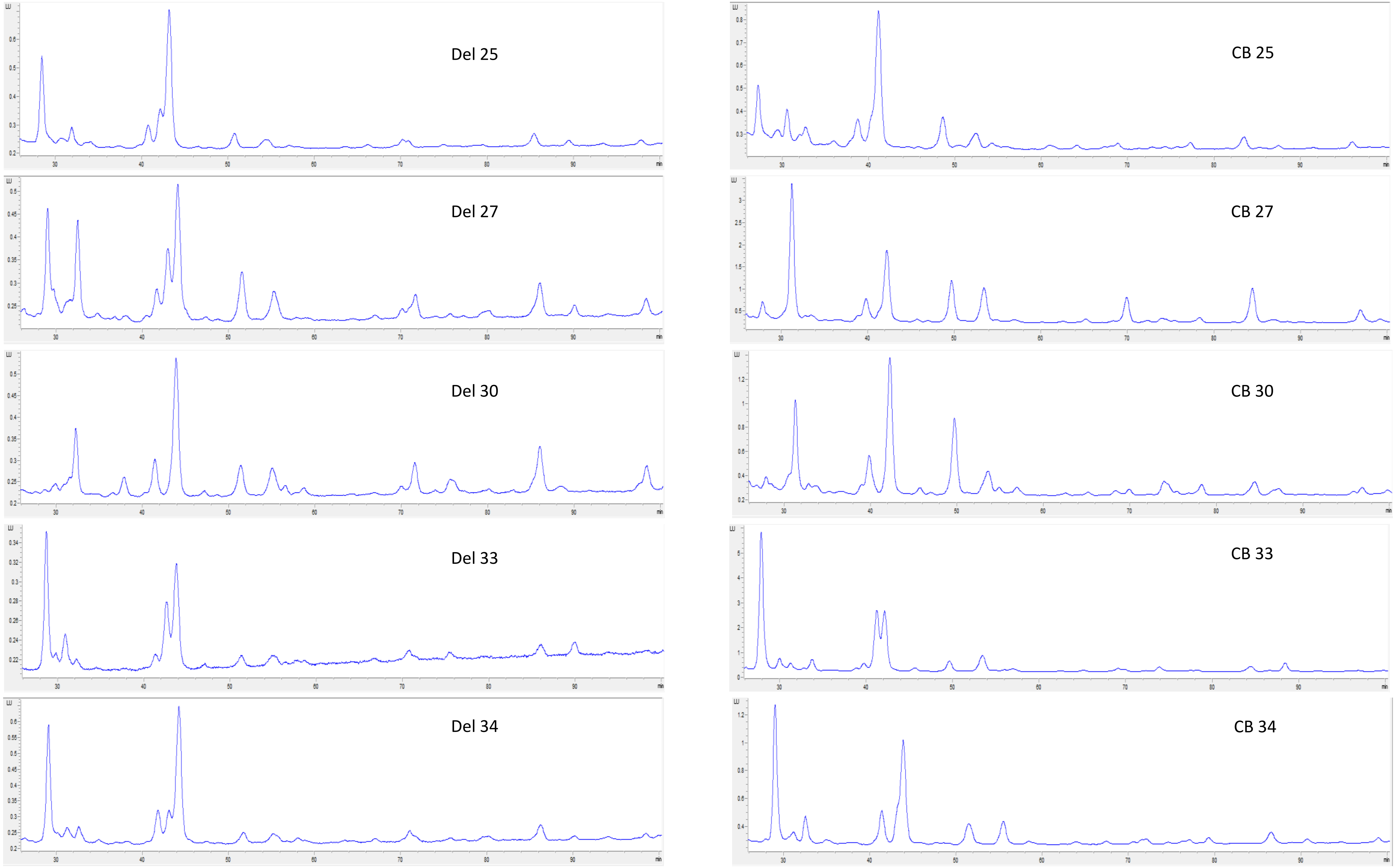
Del 25
CB 25
Del 27
CB 27
Del 30
CB 30
Del 33
CB 33
Del 34
CB 34

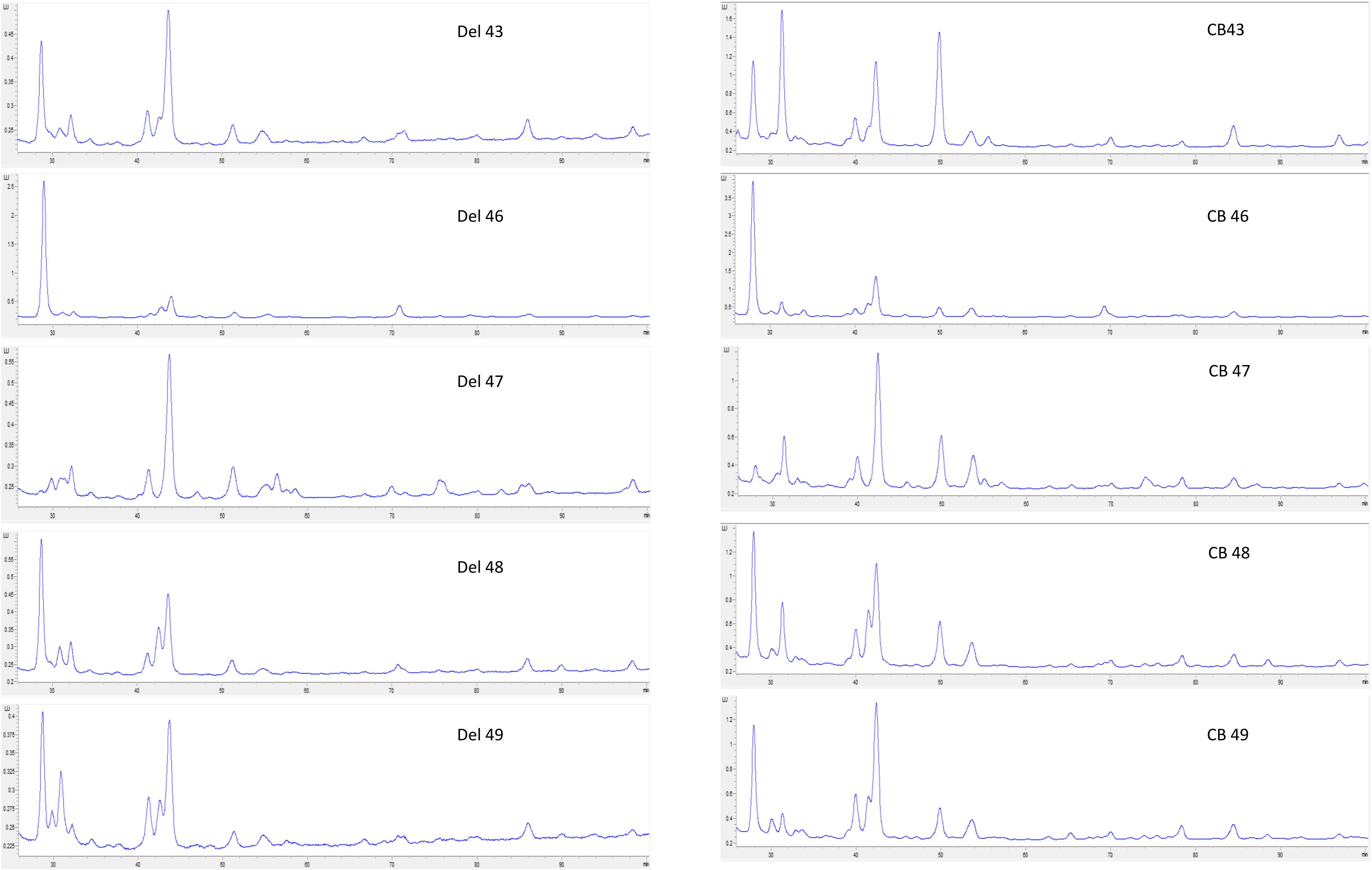
Del 43
CB43
Del 46
CB 46
Del 47
CB 47
Del 48
CB 48
Del 49
CB 49

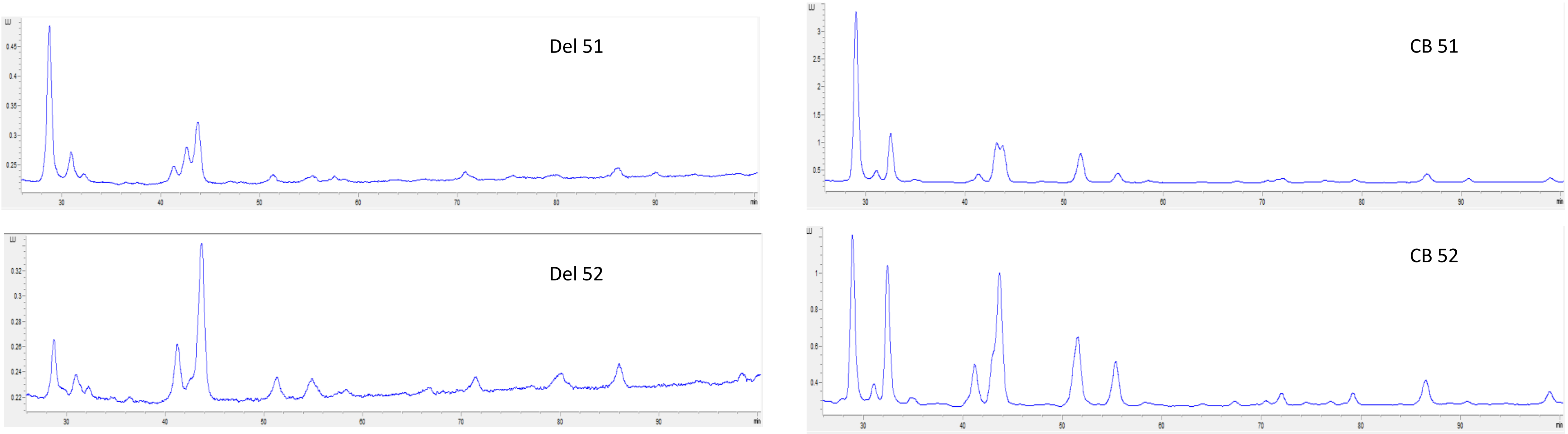
Del 51
CB 51
Del 52
CB 52

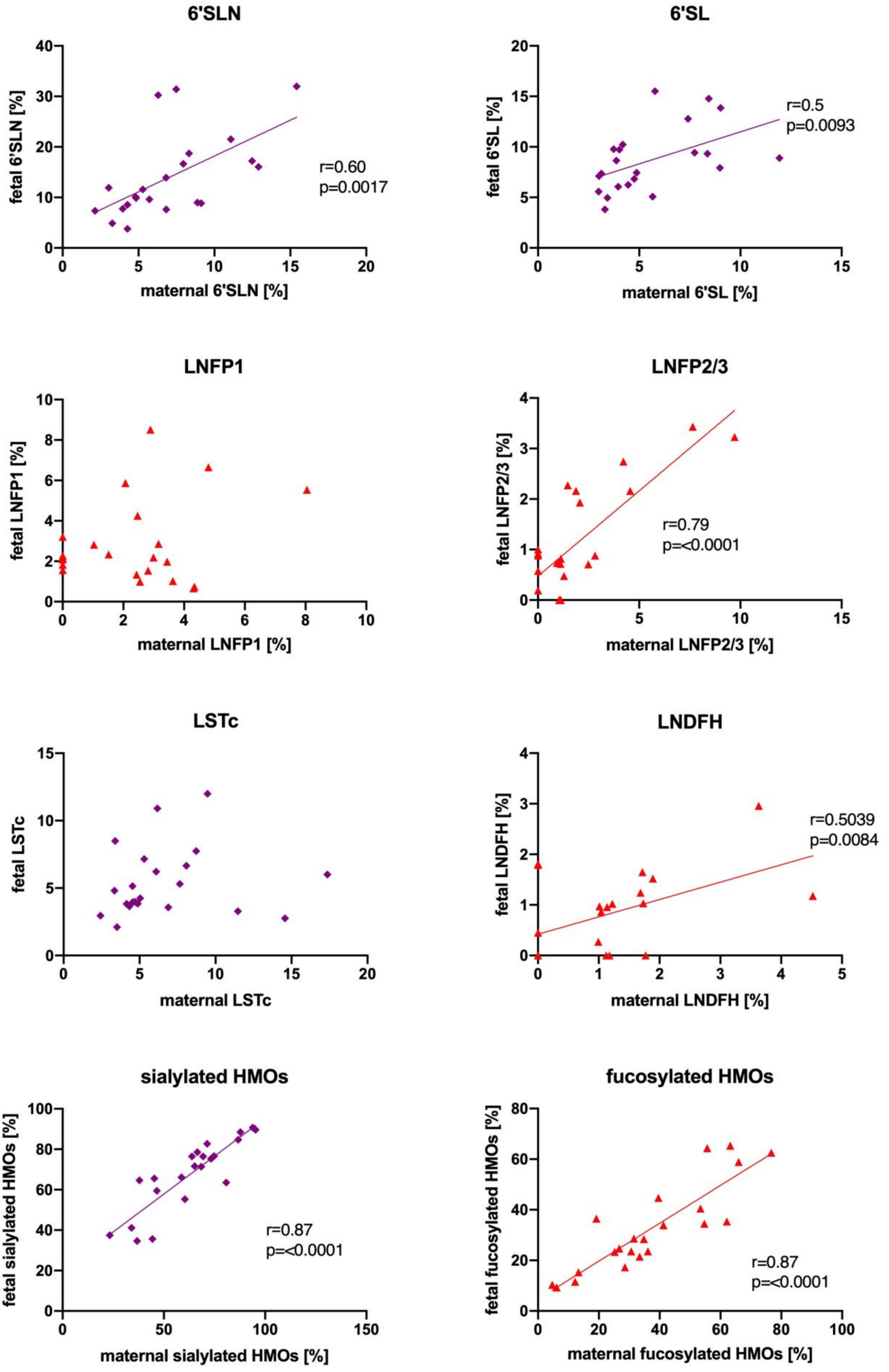
6'SLN
fetal 6'SLN [%]
maternal 6'SLN [%]
r=0.60
p=0.0017
6'SL
fetal 6'SL [%]
maternal 6'SL [%]
r=0.5
p=0.0093
LNFP1
fetal LNFP1 [%]
maternal LNFP1 [%]
LNFP2/3
fetal LNFP2/3 [%]
maternal LNFP2/3 [%]
r=0.79
p=<0.0001
LSTc
fetal LSTc
maternal LSTc
LNDFH
fetal LNDFH [%]
maternal LNDFH [%]
r=0.5039
p=0.0084
sialylated HMOs
fetal sialylated HMOs [%]
maternal sialylated HMOs [%]
r=0.87
p=<0.0001
fucosylated HMOs
fetal fucosylated HMOs [%]
maternal fucosylated HMOs [%]
r=0.87
p=<0.0001
